# Increased NO bioavailability in aging male rats by genistein and exercise training: using 4, 5-diaminofluorescein diacetate

**DOI:** 10.1186/1477-7827-7-93

**Published:** 2009-09-07

**Authors:** Sukanya Eksakulkla, Daroonwan Suksom, Prasong Siriviriyakul, Suthiluk Patumraj

**Affiliations:** 1 Inter-department of Physiology, Graduate School, Chulalongkorn University, Bangkok 10330, Thailand; 2 School of Sports Science, Chulalongkorn University, Bangkok 10330, Thailand; 3 Department of Physiology, Faculty of Medicine, Chulalongkorn University, Bangkok 10330, Thailand

## Abstract

**Background:**

Several kinds of anti-oxidants have drawn a lot of intension for their benefits on vascular protection. In addition, it has been demonstrated that exercise training could improve endothelial function by up-regulating endothelial nitric oxide synthase (eNOS) protein. Therefore, the present study aims to investigate the effects of genistein, a potent phyto-antioxidant, and exercise training on age-induced endothelial dysfunction in relation to NO bioavailability using in situ NO-sensitive fluorescent dye detection.

**Methods:**

Male Wistar rats (20-22-month old) were divided into four groups: aged rats treated with corn oil, (Aged+Veh, n = 5), aged rats treated with genistein (Aged+Gen, n = 5, (0.25 mg/kg BW/day, s.c.)), aged rats with and without exercise training (Aged+Ex, n = 5, swimming 40 min/day, 5 days/week for 8 weeks) (Aged+Without-Ex, n = 5). Cremaster arterioles (15-35 micrometer) were visualized by fluorescein isothiocyanate labeled dextran (5 microgram/ml). The vascular response to acetylcholine (Ach; 10^-5^M, 5 ml/5 min) was accessed after 1-min norepinephrine preconstriction (10 micro molar). To determine NO bioavailability, the Krebs-Ringer buffer with 4, 5-diaminofluorescein-diacetate (3 micro molar DAF-2DA), and 10 micro- molar Ach saturated with 95%N_2 _and 5%CO_2 _were used. Changes of DAF-2T-intensities along the cremaster arterioles were analyzed by the Image Pro-Plus Software (Media Cybernatics, Inc, USA). Liver malondialdehyde (MDA) level was measured by thiobarbituric acid reaction and used as an indicator for oxidative stress.

**Results:**

The results showed that means arterial blood pressure for both Aged+Gen and Aged+Ex groups were significantly reduced when compared to the Aged groups, Aged+Veh and Aged+Without-Ex (P < 0.05). Among the treated groups, Ach-induced vasodilatation were significantly increased (P < 0.05) and was associated with increased NO-associated fluorescent intensities (P < 0.05). On the other hand, MDA levels were significantly reduced (P < 0.05) when Aged+Veh was compared to Aged+Without-Ex.

**Conclusion:**

These findings showed that genistein and exercise training could improve age-induced endothelial dysfunction and is related to the increased NO bioavailability.

## Background

Aging is a dependent risk factor for the development of vascular diseases in association with a progressive endothelial dysfunction [1,1]. It is believed that reduced basal endothelium-derived vasodilators, such as nitric oxide (NO), contribute to the age-related increase in peripheral vascular resistance and systemic hypertension [3,3]. From epidemiological and experimental studies, they have shown that an increased superoxide (O_2_^-^) production with advancing age causes oxidative stress and leads to the development of endothelial dysfunction [5-10]. Superoxide (O_2_^-^) is a free radical that can rapidly scavenge NO directly, and this age-induced oxidative stress may contribute to the reduction of NO bioavailability [11]. Therefore, several studies have investigated the roles of phyto-substances with anti-oxidative properties and their benefits as vasculoprotective agents. As of note, especially in East Asian population, high plasma concentration of genistein, a soy product, contributes to a strikingly low incidence of atherosclerosis and coronary heart disease [12-14]. It has been reported that genistein, a phytoestrogen acting as estrogenic cardioprotector [13], could enhance coronary vasoreactivity and inhibit the oxidative stress [15-17]. Genistein, an anti-oxidant, could produce acute NO-dependent vasodilatation in the forearm vasculature of men and women with potency similar to that of 17β-estradiol [18-22]. Moreover, aerobic exercise training appears to reverse old age-associated reductions in endothelium-dependent vasodilatation in humans [2,23,24]. Therefore, we evaluate the effects of genistein and exercise training on protecting endothelial cells against age-induced oxidative stress by using a fluorescent indicator-diaminofluorescein (DAFs). This technique allowed us to examine the *in situ *release of NO from cremasteric endothelial cells after acetylcholine activation.

## Methods

### Animal preparation

The present study was conducted in accordance with the guidelines for animal experimentation of the National Research Council of Thailand and approved by Ethical Committee, Faculty of Medicine, Chulalongkorn University.

Male Wistar rats aged 20-22 months (n = 20) were used in this study. The animals were obtained from National Laboratory Animal Center, Salaya Campus, Nakhonprathom, Thailand, and housed in the animal laboratory center at the Faculty of Medicine, Chulalongkorn University until they were used. The rats were fed with the standard chow and drank tap water *ad libitum*. The room was temperature controlled at 25°C, and has a 12:12-light-dark cycle. The old rats were randomly divided into four groups: aged rats treated with corn oil (Aged+Veh (n = 5); Sigma-Aldrich Co., USA), aged rats treated with genistein (Aged+Gen, (n = 5); Sigma-Aldrich Co., USA, 0.25 mg/kg BW/day, s.c.) [19], and aged rats with exercise training (Aged+Ex (n = 5)). Aged+Gen rats received genistein injection every day for 8 weeks. For the Aged+Ex group, swimming exercise training protocol was conducted in 2 phases, adaptation and training. The adaptation phase consisted of the first 5 days of training. On the first day, the animals exercised in a round plastic tank (diameter = 38.5 cm, depth= 35 cm, water temperature about 34-36°C) for 10 minutes. The exercise period was extended by 10 minutes every day until the rats could swim for 40 minutes. The training phase consisted of swimming 40 min/day, 5 days/week for a total of 8 weeks [25]. Swimming exercise was selected because it did not cause foot injuries, and is physically less traumatic for the animal. In the Aged+Without-Ex group (n = 5), the rats were immersed in water as to make them wet for 30 min/day, 5 day/week for a total of 8 weeks [25]. As of note, in this study, male Wistar rats aged 4-6 months (n = 5) were used as controls.

On week 8, all rats were anesthetized intraperitoneally with pentobarbital sodium (50 mg/kg BW). After tracheotomy, a polyethylene tube was inserted into the carotid artery to measure the arterial blood pressure. The jugular vein was cannulated for fluorescence tracer. *In vivo *microcirculatory observations were performed in the cremaster muscle according to the methods described by Gavins et al. (2004) [26]. The cremaster muscle was carefully spread over a chamber that was continuously perfused with 37°C Krebs-Ringer buffer (composition in mmol/L: 135.7 NaCl, 4.7 KCl, 2.52 CaCl_2_, 1.18 KH_2_PO_4_, 1.64 MgSO_4_.7H_2_O, and 7.14 NaHCO_3_) at pH 7.4 and equilibrated with 5%CO_2_-95%N_2_. The rate of perfusion was kept constant at 2 ml/min [26].

### Arteriolar response to acetylcholine and sodium nitroprusside

The second or third-order cremasteric arterioles (15 to 35 μm in diameter) were labeled with 5% fluorescein isothiocyanate-labeled dextran (FITC-dextran 250, 5 μg/ml; Sigma-Aldrich Co., USA) which was injected into the jugular vein. After the arterioles were pre-constricted with norepinephrine (10^-5^M NE; 0.1 ml/min), it was later dilated by applying acetylcholine (10^-5^M Ach; 5 ml/5 min) topically. The changes of vascular diameters were recorded real time throughout the experiment with a black and white video monitor (Sony, GM-1411 QM) and an epi-illumination fluorescence videomicroscopy system (Optiphot 2, Nikon, Japan) equipped with a 100 W mercury lamp, real time CCD camera (Hamamatsu C2400, Japan), a video recorder (VC-S5, Sharp, Japan) with a video timer (VTG-33, For-A, Japan) and a 20 × objective lens (CF Plan Fluor, Nikon, Japan). Cremasteric arteriolar diameter was measured by using the software (Image-Pro Plus; Media Cybernatics, Inc, USA). The arteriolar diameter was calculated by averaging three measurements obtained from three different video frames using the same reference point as a marker for measuring each vessel in each frame. Arteriolar diameters were measured five minutes after Ach administration. Vasodilatation responses were expressed as the percentage of maximal relaxation after norepinephrine (NE; 10^-5^M) preconstriction.

Under the same protocol, after the arteriole was selected, it was washed by Krebs-Ringer solution (pH 7.4) until its diameter was returned to normal. The SNP (10^-5^M; 5 ml/5 min), an endothelium-independent vasodilator, was applied topically on the arterioles after it was pre-constricted with NE. Vasodilatation responses were expressed as the percentage of maximal relaxation after norepinephrine (NE; 10^-5^M) preconstriction.

### Direct detection of NO production

On the experiment day, the cremaster microcirculation was observed under intravital fluorescent video microscope with a 20 × objective lens and a 10 × eyepieces. To visualize microvascular distribution of NO, diaminofluorescein-2 (DAF-2DA, Cayman Chemical Company, Michigan, USA), a NO-sensitive fluoroprobe, was used. After the cremaster was superfused with Krebs- Ringer buffered solution containing 3 μM DAF-2DA and 10^-5^M Ach, NO levels from Ach-activated endothelial cells were analyzed at two different time points, 0 min (I_0 min_) and 20 mins (I_20 min_) [27,27].

DAF-2DA can readily enter the cells and hydrolyzed by cytosolic esterase to DAF-2, which is trapped inside the cells. In the presence of NO, the relatively non-fluorescent DAF-2 is converted into a highly green fluorescent triazole form, DAF-2T (shown in diagram below). Thus the increases in DAF-2T fluorescent intensity represent of the cremasteric microcirculation would indicate an elevation of nitric oxide having by an excitation and emission wavelengths of 488 and 538 nm, respectively [27,27,29].



**Figure 1:**
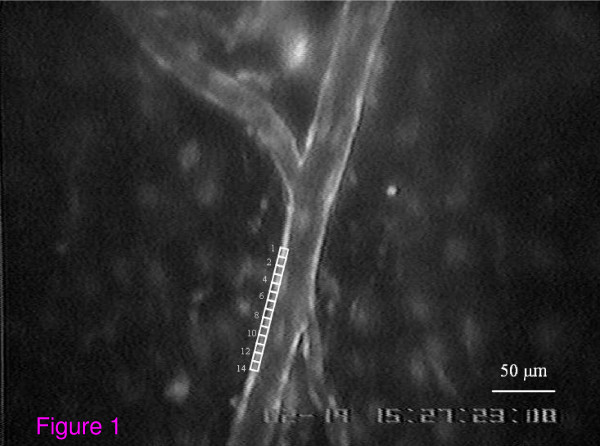
**This figure showed a videoimage of NO-associated fluorescent intensity taken from cremasteric arteriole of a young rat by using 20 ×-objective lenses**. From 14 frames of working windows, the Histogram Menu in Image Program Software was used to define the mean value of fluorescent intensity of each window, and then the averaged intensity was calculated for all 14 small working window frames (7 × 3 μm^2 ^each window). The intensity difference (I_diff_) between the averaged intensities obtained at 20 min (I_20 min_) and at baseline (I_0 min_) were calculated and represented by the percentage change in intensity from its baseline value, using the following equation: %Change of NO-associated fluorescent intensity = (I_20 *min*_- I_0 *min*_/I_0 *min*_)×100



The cremasteric microcirculation was epi-illuminated having an excitation wavelength of 488 nm and emission wavelength of 538 nm. The microscopic field containing arterioles (15 to 35 μm in diameter) sharing the same focusing plane were selected and recorded for further analysis using Image Pro-Plus V. 5 software. (Media Cybernatics, Inc, USA). From fourteen small working window frames (7 × 3 μm^2 ^each window), the fluorescent intensity of each arteriolar vascular wall was averaged (Figure 1). Assuming that DAF-2T intensity is linearly related to the intracellular NO content, the difference in fluorescent intensity between I_0 min _and I_20 min _was calculated according to the following equation and represented as the percent increase in NO released during the first 20 minutes:

### Measurement of metabolic parameters

At the end of each experiment, the blood sample of each rat was collected to determine the androgen level. Immediately after that, the liver was perfused with ice-cold phosphate buffer saline (PBS) at pH 7.4 and excised. All fat and fibrous tissues on the liver were removed before it was weighed. The liver was kept at -80°C until malondialdehyde (MDA) analyses were done. MDA was performed by using the thiobarbituric acid reaction as described by Ohkawa et al., 1979 [31].

### Statistical analyses

Data are expressed as means ± SD. For comparison among groups of animals, one-way analysis of variance (one-way ANOVA) and a two-sided alpha level of 0.05 adjusted by Tukey's procedure for multiple comparisons were used. *P *< 0.05 was considered statistically significant.

## Results

### Physiological characteristics

In Table 1, the results showed that means arterial pressure (MAP) for age-groups (Aged+Veh; 144.68 ± 5.93 mmHg, and Aged+Without-Ex; 144.20 ± 18.62) were significantly higher than the young group (4-6-mo-old; 121.55 ± 3.43 mmHg) (*P *< 0.01). However, MAP from both Aged+Gen (124.58 ± 2.41 mmHg) and Aged+Ex rats (124.20 ± 5.31 mmHg) reduced significantly when it was compared to their age- matched control groups (*P *< 0.05). Testosterone levels and the ratios of seminal vesicle/body weights were significantly decreased in Aged+Veh (0.71 ± 0.74 ng/mL and 0.0023 ± 0.0005) and Aged+Without-Ex groups (0.67 ± 0.77 ng/mL and 0.0023 ± 0.0018) when compared to the young group (2.01 ± 1.31 ng/mL and 0.0044 ± 0.0008) (*P *< 0.05). Genistein supplementation showed no effects on both testosterone level and the ratio of seminal vesicle/body weight. However, in Aged+Ex group, the ratio of seminal vesicle/body weight was significantly higher than the Aged+Without-Ex group (Aged+Ex = 0.0042 ± 0.0006, Aged+Without-Ex = 0.0023+0.0018) (*P *< 0.05). This may be due to the effect of exercise training on the fat composition, because there were no significant differences between seminal vesicle weight of the Aged+Ex and Aged+Without-Ex groups.

**Table 1:** Means ± SD of physiological and biochemical characteristics of young, aged rats with vehicle (Aged+Veh), genistein (Aged+Gen), without exercise training (Aged+Without-Ex), and exercise training (Aged+Ex).

**Group**	**MAP (mmHg)**	**Testosterone (ng/mL)**	**Seminal vesicle weight (g)**	**Seminal vesicle weight/body weight**	**MDA level of liver (μmol/g wet wt.)**
Young *(n = 5)*	121.55 ± 3.43	2.01 ± 1.31	2.09 ± 0.38	0.0044 ± 0.0008	2.42 ± 0.48
Aged+Veh *(n = 5)*	144.68 ± 5.93**	0.71 ± 0.74*	1.74 ± 0.33	0.0023 ± 0.0005*	4.81 ± 1.17*
Aged+Gen *(n = 5)*	124.58 ± 2.41^#^	1.13 ± 0.73	2.49 ± 0.67	0.0036 ± 0.0008	2.53 ± 0.59^#^
Aged+Without-Ex *(n = 5)*	144.20 ± 18.62**	0.67 ± 0.77*	1.54 ± 1.31	0.0023 ± 0.0018*	4.33 ± 2.00*
Aged+Ex *(n = 5)*	124.20 ± 5.31^†^	0.34 ± 0.09	2.87 ± 0.29	0.0042 ± 0.0006^†^	2.51 ± 0.49^†^

*P < 0.05 compared with young group, **P < 0.01 compared with young group, ^#^*P *< 0.05 compared with Aged+Veh group, ^†^*P *< 0.05 compared with Aged+Without-Ex group.

### Levels of liver malondialdehyde

Indicators of oxidative stress, liver-malondialdehyde (MDA), of Aged+Veh (4.81 ± 1.17 μmol/g liver wet weight) and Aged+Without-Ex (4.33 ± 2.00 μmol/g liver wet weight) were significantly higher when compared to the young group (2.42 ± 0.48 μmol/g liver wet weight) (*P *< 0.05). But the MDA levels of two treated groups, Aged+Gen (2.53 ± 0.59 μmol/g liver wet weight) and Aged+Ex (2.51 ± 0.49 μmol/g liver wet weight), were significantly lower when compare to their age-matched controls (*P *< 0.05) (Table 1).

### Acetylcholine-induced arteriolar response

**Figure 2:**
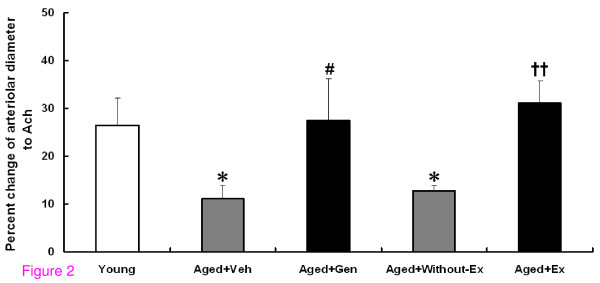
**The percentage of increase in arteriolar diameters induced by Ach for young, aged rats with vehicle (Aged+Veh), genistein (Aged+Gen), without exercise training (Aged+Without-Ex), and exercise training (Aged+Ex)**. *P < 0.05 compared with young group, ^#^*P *< 0.05 compared with Aged+Veh group, ^††^*P *< 0.01 compared with Aged+Without-Ex group.

As shown in Figure 2, arteriolar dilatation to Ach was significantly impaired in Aged+Veh (11.07 ± 2.83%) when it was compared to the young group (26.51 ± 5.60%) (*P *< 0.05). Interestingly, the dilatory responses of the arterioles to Ach significantly increased in both Aged+Gen (27.41 ± 8.75%) and Aged+Ex (31.15 ± 4.58%) groups when compared to their age-matched controls (*P *< 0.05 and *P *< 0.001, respectively).

### Sodium nitroprusside-induced arteriolar response

**Figure 3:**
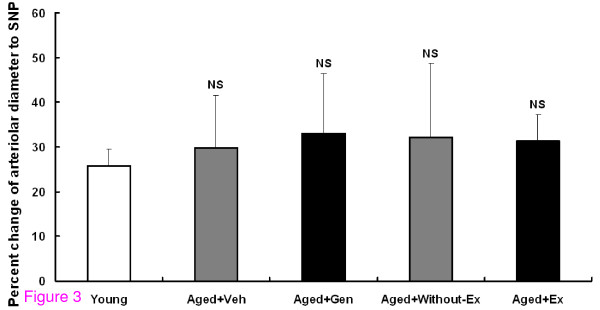
**The percentage change of arteriolar diameters induced by SNP for young, aged rats with vehicle (Aged+Veh), genistein (Aged+Gen), without exercise (Aged+Without-Ex) and exercise (Aged+Ex)**. No significantly difference as compared to young group. No significantly difference as compared to Aged groups.

To ensure that impairment of arteriolar dilatation to Ach did not interfere with the function of the smooth muscle cells, the vasodilatory response to endothelium-independent vasodilatation was examined by using SNP (10^-5^M). The results showed that there was no significant difference among the young (25.88 ± 3.70%), Aged groups (Aged+Veh, (29.79 ± 11.71%), and Aged+Without-Ex groups (32.15 ± 16.56%)) (Figure 3).

### Direct detection of NO production

**Figure 4:**
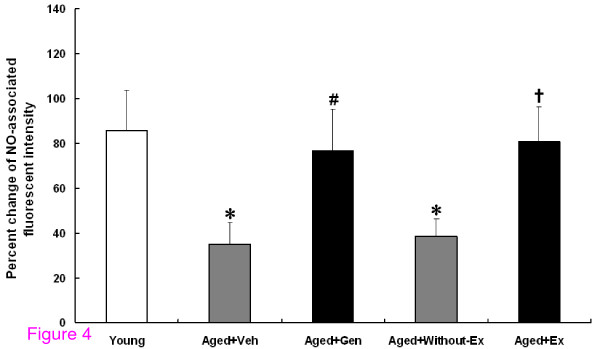
**The percentage change of NO-associated fluorescent intensity for young, aged rats with vehicle (Aged+Veh), genistein (Aged+Gen), without exercise (Aged+Without-Ex) and exercise (Aged+Ex)**. *P < 0.05 compared with young group, ^#^*P *< 0.05 compared with Aged+Veh group, ^†^*P *< 0.05 compared with Aged+Without-Ex group.

In Figure 4, the NO production was significantly lower in the aged groups (Aged+Veh (35.01 ± 9.71%) and Aged+Without-Ex (38.47 ± 7.84%) when compared to the young group (85.74 ± 17.99%) (*P *< 0.001). Interestingly, both Aged+Gen (76.64 ± 18.52%) and Aged+Ex (80.86 ± 15.62%) groups had a significant enhancement of the NO level when compared to their age-matched controls (Aged+Veh and Aged+Without-Ex) (*P *< 0.05). It is noted that genistein and exercise training could enhance NO bioavailability in aging rats.

**Figure 5:**
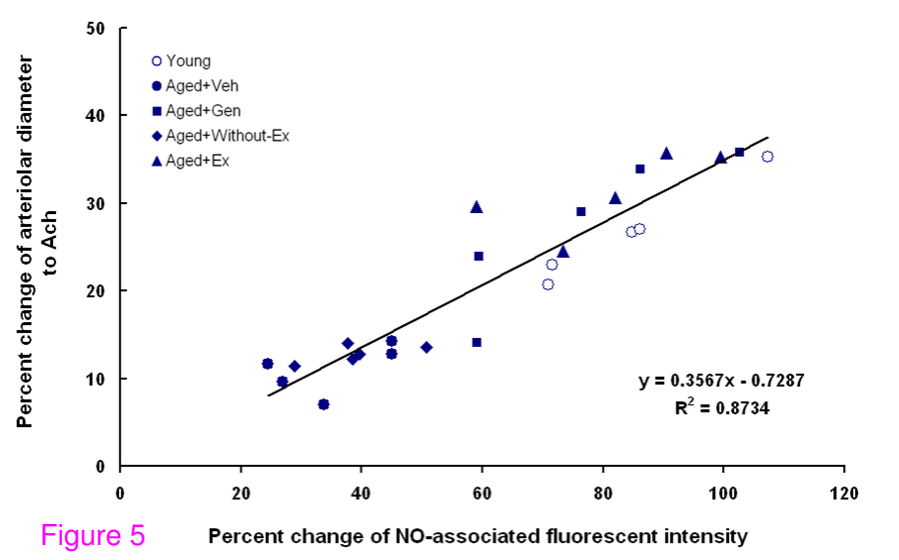
**The correlations between percent changes of NO-associated fluorescent intensity and the percent arteriolar diameter changes were examined by using Pearson's Correlation and the best-fitting linear regression**. All data were taken from values of each groups: young, aged+vehicle (Aged+Veh), aged+genistein (Aged+Gen), aged+without exercise (Aged+Without-Ex) and aged+exercise (Aged+Ex). (Pearson's correlation = 0.87, *P *< 0.01).

In Figure 5, linear regression was performed to establish the relationship between the Ach-induced increase in arteriolar diameters and the percent increased in NO-associated fluorescent intensity for the young, Aged+Veh, Aged+Gen, Aged+Without-Ex and Aged+Ex groups. The linear equation obtained was: y = 0.3567x - 0.7287, R^2 ^= 0.87, (*P *< 0.01), where x is the mean of NO-associated fluorescent intensity and y is the mean percentage of diameter change.

## Discussion

In the present study, we have shown that soy phytoestrogen, genistein, and 8-week exercise training could reduce the age-induced cardiovascular abnormalities, such as hypertension and age-induced endothelial dysfunction. These treatments can increase NO bioavailability to have a linear correlation with endothelial-dependent vasodilatation improvement.

Since it is well known that age-induced oxidative stress can cause abnormal vascular functions by enhancing resistance to blood flow, hence, in this study, we measured the MDA levels in liver of the rats from the age-groups. The condition of the age-induced oxidative stress was indicated by an elevated MDA level which can contribute to the sequential process, age-induced endothelial dysfunction [8,8]. In the report of Taddei et al. [9], it has been confirmed that the response to acetylcholine declined with age. Moreover, it has been suggested that the phenomenon of age-induced endothelial dysfunction is widespread and applies to the whole circulation [32]. For that reason, it is possible to increase the total peripheral vascular resistance and MAP in the aged group.

The results from the MDA levels showed that long-term treatment of genistein can scavenge the age-induced excess free radicals, especially superoxide anions which can directly damage molecules of protein, lipid, and DNA as which leads to cell dysfunction. Our observation of increased Ach-induced vasodilatation in Aged+Gen group confirms that anti-oxidant agent can prevent age-induced endothelial dysfunction. This ability may enhance endothelial-dependent vasodilatation by protecting NO from direct interaction with the superoxide radical. Since NO bioavailability is associated with endothelium-dependent vasodilatation, the greater NO bioavailability level, therefore, a more pronounced endothelium-dependent vasodilatation effect will be observed.

Furthermore, many investigators are interested in genistein's mechanism and how it is able to increase NO bioavailability [21,21]. For example, Walker et al. showed that genistein, 17β-estradiol, can produce acute NO-dependent vasodilatation which may affect endothelial nitric oxide synthase (eNOS) activity [22]. On the other hand, it has been shown that genistein has a similar affinity as estrogen because of the novel ER-β present in the vasculature [21]. The ER-β in endothelial cell has been shown to stimulate NO-production also known as the non-genomic effect of estrogen [33]. Interestingly, it has been shown that acute topical application of genistein mediated vasodilatation via prostacyclin (PGI_2_) and NO productions [34].

Yet, other investigations indicated that an acute action of genistein-induced endothelial NO production was mediated by another pathway of protein kinase A (PKA) which is unrelated to the estrogenic effect [35]. They also suggested that this pathway may be crucial in protecting the cardiovascular effects seen in soy phytoestrogens. For example, Liu et al. (2004) [35] demonstrated that 1-μM genistein could directly activate eNOS in intact bovine aortic endothelial cells and endothelial cells from human umbilical vein over an incubation period of 10 minutes. They also proposed that these effects were mediated by PKA and were unrelated to the estrogenic effect. In another report, Si et al. (2008) showed that genistein had a direct genomic effect on the vascular wall causing an increased eNOS expression and NO synthesis in spontaneously hypertensive rat model [36].

Our result (Table 1) confirmed that both testosterone levels and ratios of seminal weights were significantly decreased by aging. Genistein supplementation and exercise training did not affect those parameters. Other studies have reported similar findings that low doses of phytoestrogens do not have any effects on reproductive functions of either males or females [37,37]. However, the increased seminal vesicle/body weight ratio in Aged+Ex group may have been from the metabolic effect of exercise training on fat composition leading to weight loss in this group.

Aside from that, our findings indicated that genistein and exercise training could protect endothelial functions from age-induced dysfunction. A significant reduction in MDA levels in Aged+Ex group was observed when compared to the Aged+Without-Ex group (Table 1). These results confirmed the effectiveness of our training protocol, 40 min/day, 5 day/week for a total of 8 weeks, in reducing peripheral vascular resistance in aged male rat. The following might help explain the benefits of long-term exercise training: 1) reduction of age-induced oxidative stress by increasing the enzymatic anti-oxidants expression, and 2) an increase in shear-stress mediated eNOS activity [39]. Also Ahmadiasl and colleagues (2007) showed that long-term endurance training could increase superoxide dismutase (SOD) activities in rat myocardium [40]. There are several approaches that may explain how exercise training can enhance endothelial function, yet the effects of endurance exercise training on the oxidative status and antioxidant defense system remain unclear. Discrepancies are due to different training protocols used and, therefore, caution is warranted in interpreting these results.

Nevertheless, in our study, a moderate exercise training program consisting of 8 weeks is sufficient enough to reduce age-induced endothelium dysfunction. It appears to us that exercise training has the same effects as seen in genistein because of its ability to protect NO bioavailability in the Aged group and enhance endothelium-dependent vasodilatation. The reason for this may be that exercise training improves endothelial function and provides cardioprotective benefits.

Additional analysis using an *in vitro *study, Kashiwagi et al. (2002) showed a linear relationship between the gray levels of fluorescence intensities and DAF-2T concentrations (between 10 nmol/L and 1 μmol/L) [27]. For this reason, we chose to further investigate whether there are any correlation between NO-associated fluorescent intensity and the Ach-evoked vasodilatation in all five groups. Our correlation results between the means percent changes of NO-associated fluorescent intensity and the mean percent of arteriolar diameter changes from all 5 groups were significantly correlated and are shown in Figure 5 (Pearson's correlation = 0.87, *P *< 0.01). These findings suggested that Ach-induced arteriolar dilatation could be restored by using genistein and exercise training to increase intra-endothelial NO bioavailability.

The results obtained showed an increase of NO bioavailability after utilizing two interventions. Both of these interventions have multiple functions effecting eNOS expression, eNOS activity, and eNOS cofactor; tetrahydrobiopterin (BH_4_).

Both interventions can increase eNOS expression through both direct and indirect pathways [35,36,41,42]. Similarly, Tanabe and his colleagues (2003) reported that the 8-week exercise swimming could up-regulate eNOS expression in the aorta in aged rats [41]. Along the same lines, Sindler et al. (2009) suggested that exercise training can restore the important eNOS cofactor, tetrahydrobiopterin (BH_4_), content and then leads to enhance flow-stimulated NO availability in old rats [42].

Our findings parallel previous research showing NO bioavailability enhances endothelium-dependent vasodilatation. Our results may be the first *in vivo *evidence of genistein and exercise training in protecting endothelial cells against age-induced oxidative stress by using a fluorescent indicator-diaminofluorescein (DAFs). The *in situ *release of NO from cremasteric endothelial cells after acetylcholine activation was examined in both genistein and exercise training groups. The effect of genistein and exercise training on increased NO bioavailability may be associated with multiple direct and indirect pathways and additional studies are needed to confirm these results.

## Conclusion

The findings indicated that long-term genistein treatment and exercise training could improve age-induced endothelial dysfunction in cremasters and increase NO bioavailability in aging male rats.

## Competing interests

The authors declare that they have no competing interests.

## Authors' contributions

SE assisted the co-authors in the design of study. The experiments were carried out by SE. SP, PS and DS supervised the experiments, interpreted and analyzed the data. SE prepared the initial draft of the manuscript and SP mindfully read and revised the manuscript. All authors have read and approved the manuscript.
